# Downregulation of the Taurine Transporter TauT During Hypo-Osmotic Stress in NIH3T3 Mouse Fibroblasts

**DOI:** 10.1007/s00232-012-9416-8

**Published:** 2012-03-02

**Authors:** Daniel Bloch Hansen, Martin Barfred Friis, Else Kay Hoffmann, Ian Henry Lambert

**Affiliations:** Department of Biology, Section of Cellular and Developmental Biology, University of Copenhagen, The August Krogh Building, Universitetsparken 13, 2100 Copenhagen Ø, Denmark

**Keywords:** NADPH oxidase, Hyponatremia, Osmolyte transport, Hypo-osmolal

## Abstract

The present work was initiated to investigate regulation of the taurine transporter TauT by reactive oxygen species (ROS) and the tonicity-responsive enhancer binding protein (TonEBP) in NIH3T3 mouse fibroblasts during acute and long-term (4 h) exposure to low-sodium/hypo-osmotic stress. Taurine influx is reduced following reduction in osmolarity, keeping the extracellular Na^+^ concentration constant. TonEBP activity is unaltered, whereas TauT transcription as well as TauT activity are significantly reduced under hypo-osmotic conditions. In contrast, TonEBP activity and TauT transcription are significantly increased following hyperosmotic exposure. Swelling-induced ROS production in NIH3T3 fibroblasts is generated by NOX4 and by increasing total ROS, by either exogenous application of H_2_O_2_ or overexpressing NOX4, we demonstrate that TonEBP activity and taurine influx are regulated negatively by ROS under hypo-osmotic, low-sodium conditions, whereas the TauT mRNA level is unaffected. Acute exposure to ROS reduces taurine uptake as a result of modulated TauT transport kinetics. Thus, swelling-induced ROS production could account for the reduced taurine uptake under low-sodium/hypo-osmotic conditions by direct modulation of TauT.

**Electronic supplementary material:**

The online version of this article (doi:10.1007/s00232-012-9416-8) contains supplementary material, which is available to authorized users.

## Introduction

The ability to restore cell volume following osmotic perturbation is pivotal for cell function, and we have recently reviewed the intracellular signaling events evoked by cell swelling and cell shrinkage, as well as the biophysical and pharmacological characteristics of volume-sensitive transporters for organic and inorganic osmolytes (Hoffmann et al. 2009; Lambert et al. 2008). Mammalian cells restore their cell volume following osmotic perturbation; i.e., KCl, organic osmolytes (amino acids/sugars), and water are released to or taken up from the extracellular compartment following cell swelling and cell shrinkage, respectively (Hoffmann et al. 2009). The organic acid taurine is quantitatively an important osmolyte in mammalian cells, and even though taurine is mainly recognized through its contribution to the cellular pool of organic osmolytes, it has in recent years turned out that taurine modulates multiple cellular functions through stabilization of membrane integrity, modulation of ion channel activity, shifts in membrane phospholipid content and, hence, activity of enzymes embedded in the membrane (Jong et al. 2010) or elimination of reactive oxygen species (ROS) and thereby limitation in lipid peroxidation (Goodman et al. 2009). Taurine is taken up from the extracellular compartment via the Na^+^-dependent taurine transporter TauT and released via a volume-sensitive leak pathway which is permeable to a range of organic osmolytes (Lambert and Hansen 2011; Hall et al. 1996; Lambert 2004). Expression of TauT is regulated by p53, c-Jun, WT1 (Wilms tumor gene 1) and TonEBP (Chesney et al. 2010; Lambert 2004), whereas TauT activity is acutely controlled through direct phosphorylation/dephosphorylation of TauT and/or a regulator of TauT (Hansen et al. 2011; Jacobsen et al. 2008; Voss et al. 2004; Lambert 2004). The volume-sensitive leak pathway has not been cloned but is often referred to as the volume-sensitive organic anion channel (VSOAC) (Hansen et al. 2011; Lambert 2004).

ROS in limited quantities are considered essential second messengers, whereas ROS in larger quantities become harmful to cell function and cause cell damage and cell death. ROS production increases following osmotic cell swelling (Supplementary Fig. 2) (Diaz-Elizondo et al. 2006; Friis et al. 2008; Lambert 2003; Varela et al. 2004; Ørtenblad et al. 2003; Hansen et al. 2011) as well as osmotic cell shrinkage (Zhou et al. 2006; Yang et al. 2005; Eisner et al. 2006), and it has previously been shown that ROS potentiate the swelling-induced taurine release, presumably through inactivation of protein tyrosine phosphatases and, hence, an increase in the phosphorylation of tyrosine residues of enzymes involved in the activation of the volume-sensitive taurine transporter or the transporter itself (Hansen et al. 2011; Lambert 2003). It appears that NADPH oxidases constitute the catalytic core for ROS production under hypo-osmotic conditions (Friis et al. 2008), whereas ROS under hyperosmotic conditions are of mitochondrial origin (Zhou et al. 2006). Hyperosmotically induced transcription of TauT is under the control of the tonicity-responsive enhancer binding protein (TonEBP). TonEBP is transactivated by ROS under hyperosmotic conditions (Zhou et al. 2006), whereas TonEBP mRNA is reduced and TonEBP retained in the cytoplasm under hypo-osmotic conditions (Woo et al. 2000).

Hyponatremia involves several clinical conditions that affect as much as 22% of hospitalized patients (see Loh and Verbalis 2008). Hyponatremia results in decreased sodium plasma levels from approximately 150 to <135 mM and is often associated with hypo-osmolarity caused by excessive renal water retention (Wakil et al. 2011; Upadhyay and Gormley 2011). Hyponatremia and generally cell swelling have been associated with increased oxidative stress (Barsony et al. 2011; Haussinger and Schliess 2008; Friis et al. 2008). Previous studies have demonstrated depletion of organic osmolytes, e.g., the brain taurine pool being reduced to 17% following chronic hyponatremia (Clark et al. 1996; Massieu et al. 2004). We initiated the present work to test whether hyponatremic/swelling-induced ROS production, besides initial potentiation of the swelling-induced taurine release, would modulate TauT activity directly or indirectly through TonEBP activity and TauT transcription, altering taurine uptake following hypo-osmotic hyponatremic exposure.

## Materials and Methods

### Cell Culture

NIH3T3 fibroblasts were grown at 37°C, 5% CO_2_ in 75 cm^2^ tissue culture flasks (Cellstar; Greiner Bio-One, Frickenhausen, Germany) in DMEM (335 mOsm) supplemented with 10% fetal bovine serum (FBS) and 1% penicillin/streptomycin. Cells were subcultured every 3–4 days using 0.25% trypsin in phosphate-buffered saline (PBS) containing 137 mM NaCl, 2.6 mM KCl, 6.5 mM Na_2_HPO_4_, and 1.5 mM KH_2_PO_4_. Penicillin, streptomycin, Dulbecco’s modified Eagle medium (DMEM), fetal calf serum and trypsin were from Invitrogen (Naerum, Denmark).

### Media

#### Media for Taurine Influx and Estimation of ROS

iso-osmotic DMEM (335 mOsm) contained (in mM) 1.4 CaCl_2_, 0.4 MgSO_4_, 5.4 KCl, 44 NaHCO_3_, 110 NaCl, 0.79 NaH_2_PO_4_, and 25 d-glucose supplemented with 2 ml amino acid solution (Sigma R7131; Sigma, St. Louis, MO) per liter. Low Na^+^ hypo-osmotic DMEM (200 mOsm) was obtained by reduction of NaCl to 34 mM. Low Na^+^ iso-osmotic DMEM (335 mOsm) was obtained from the low Na^+^ hypo-osmotic DMEM by supplementation with 0.85 mmol sucrose per millimole reduction in NaCl (21.8 g/l) (Hoffmann and Lambert 1983). Iso-osmotic NaCl Ringer (335 mOsm) contained (in mM) 158 NaCl, 5 KCl, 1 Na_2_HPO_4_, 1 CaCl_2_, 0.1 MgSO_4_, and 10 HEPES (*N*-2-hydroxyethyl piperazine-*N′*-2-ethanesulfonic acid). Low Na^+^ hypo-osmotic NaCl Ringer (200 mOsm) was prepared by reduction of the NaCl concentration in the iso-osmotic NaCl medium to 91 mM. Low Na^+^ iso-osmotic NaCl Ringer (335 mOsm) was prepared from low Na^+^ hypo-osmotic NaCl Ringer by supplementation with sucrose as described above for DMEM. Ringer for Na^+^ kinetic experiments was prepared by substituting *N*-methyl-d-glucamine for Na^+^.

#### Media for TonEBP/mRNA Assays

Hypo-osmotic DMEM (200 mOsm) and hyperosmotic DMEM (500 mOsm) were obtained by dilution of DMEM (iso-osmotic, Invitrogen) with buffered water (5 mM HEPES) and addition of 80 mM NaCl, respectively. All media were supplemented with 10% FBS and 1% penicillin/streptomycin.

#### Taurine Influx

NIH3T3 cells were grown to 80% confluence in six-well polyethylene dishes (9.6 cm^2^/well). Influx was estimated in cells preincubated for 4 h with the indicated DMEM medium or exposed acutely to the indicated NaCl Ringer. Prior to influx, cells were washed two times by gentle aspiration/addition of 600 μl of the respective medium/Ringer. ^3^H-taurine (Amersham, Aylesbury, UK; 629 GBq/mmol) was added to cells in well 1.5 at 0, 3, 6, 9, and 12 min, respectively (final taurine concentration 4.5 nM). At 15 min taurine uptake was terminated by removal of the extracellular medium, rapid addition/aspiration of 1 ml ice-cold MgCl_2_ (115 mM), followed by cell lysis with 200 μl 96% ethanol. The ethanol was blown off and the cellular ^3^H-taurine extracted by addition of 600 μl ddH_2_O (30 min), which was transferred to a scintillation vial for estimation of ^3^H activity (β-scintillation counting, Ultima Gold^™^; Perkin-Elmer, Waltham, MA). Each well was washed twice with ddH_2_O. The total ^3^H-taurine (cpm) taken up by the cells at a given time point was in each case estimated as the sum of ^3^H activity in the cell extract and water washouts. Uptake (cycles per minute in each well) was converted to nanomoles per gram of protein using the extracellular specific activity and the protein content (milligrams of protein per well). The latter was estimated in the sixth well by the Lowry et al. (1951) method using BSA as standard. TauT affinity for Na^+^, maximal transport rate and Na^+^:taurine stoichiometry were estimated by fitting taurine uptake at various Na^+^ concentrations in *N*-methyl-d-glucamine chloride to a Hill-type equation as previously described (Hansen et al. 2011).

#### TonEBP Activity—Luciferase Assay

The -1233-1105 TonEBP-luciferase plasmid (-1233-1105) and the -1233-1105 TonEBP-luciferase mutant plasmid (-1233-1105 M) were kind gifts from Dr. J. D. Ferraris (National Institutes of Health, Bethesda, MD). The constructs were made as outlined (Trama et al. 2000; Zhou et al. 2005). Briefly, the -1233-1105 construct contains the binding motif for TonEBP, fused upstream to the luciferase gene, whereas the mutant has a nonfunctional binding motif. The constructs were transformed into DH10α-competent cells and subsequently isolated using E.Z.N.A Fastfilter Midi Kit (Omega Bio-Tek, Norcross, GA; cat. no. D690503). NIH3T3 cells were grown to 50% confluence in a six-well dish prior to transfection. Transient transfection was performed using Lipofectamine 2000 (Invitrogen, cat. no. 11668-027) according to the manufacturer’s instructions. Briefly, 1 μg of plasmid was mixed with 5 μl Lipofectamine 2000 and 200 μl serum-free DMEM without penicillin/streptomycin and left at room temperature for 30 min. The cells in one well of the six-well dish were incubated in 1.8 ml serum-free DMEM without penicillin/streptomycin, and 200 μl transfection solution was added. The transfection medium was substituted with 2 ml of DMEM containing serum 2.5 h later. Cells were transfected 48 h before experimental use.

All luciferase measurements were performed following 4 h incubation in iso-osmotic, hyperosmotic, or hypo-osmotic DMEM. Transfected cells (-1233-1105) were lysed in 120 μl cell culture lysis buffer (Sigma, cat. no. C-4707) and left for 15 min at room temperature. Cell debris was removed and protein content estimated according to the principles of the Lowry et al. (1951) method. Luciferin assay reagent (100 μl; luciferase assay buffer plus luciferase assay substrate; Promega, Madison, WI; cat. no. E1501) was added to 20 μl cell lysates and the luciferase activity estimated with a RamCon (Birkerod, Denmark) Fluostar Optima plate reader. Luminescence was normalized by background subtraction (-1233-1105 M-transfected cells) and calculated relative to the amount of protein in the sample.

#### TauT mRNA—qPCR

NIH3T3 fibroblasts were grown to 70–80% confluence in tissue culture flasks (75 cm^2^). Cells were washed once in PBS, trypsinized and spun down (600×*g*) and total mRNA was isolated according to the manufacturer’s instructions, using the GenElute Mammalian Total RNA miniprep kit (Sigma). Total mRNA (1,500 ng) was used for cDNA synthesis using random nonamers (Sigma) and Superscript II avian reverse transcriptase (Invitrogen). cDNA was synthesized under the following conditions: 500 nM dNTP (each) and 1,500 ng total mRNA were mixed with 2.5 μM random nonamers, incubated for 10 min at 25°C, heated to 65°C for 10 min and finally transferred to ice. Reaction buffer, 200 units Superscript II (Invitrogen) and 10 μM dithiothreitol (DTT) were added to the sample, which was incubated at 25°C for 10 min, 42°C for 50 min and finally 72°C for 10 min. Following incubations, samples were transferred to ice.

qPCR was performed using the Brilliant SYBR^®^ green qPCR Master Mix (Agilent, Palo Alto, CA). Triplet measurements of TauT and β-actin expression were performed on each sample. Briefly, 25 μl reaction mixtures were made containing 112.5 ng cDNA, 1× master mix, 30 nM reference dye and 100 nM primer mix (TauT: forward 5′-ATCCTGGGCTTCATGGCACAAG-3′, reverse 5′-ATAGACCAAAAGGTGGGCAGCG-3′; β-actin: forward 5′-AGAGCTATGAGCTGCCTGAC-3′, reverse 5′-GGATGCCACAGGATTCCATAC-3′). qPCR was performed under the following conditions: 10 min at 95°C, followed by 40 cycles of 30 s at 95°C, 1 min at 60°C, 1 min at 72°C and a single final elongation step for 3 min at 72°C. The mean C_T_ value was calculated. TauT expression in each sample was calculated relative to β-actin expression to normalize differences in cDNA in each sample. Subsequently, values from each sample were calculated relative to the iso-osmotic control.

#### NOX4 Construct

Total RNA was isolated from mouse kidney renal cortex, using GenElute mammalian total RNA mini prep (Sigma, cat. no. RTN70). NOX4 was cloned using the Superscript III One-step RT-PCR system with Platinum Taq High Fidelity (Invitrogen, cat. no. 12574-030) with specific NOX4 primers (forward 5′-GAGAATTCTGGCGGTGTCCTGGAGG-3′, reverse 5′-GGGGTACCTCAGCTGAAGGATTCTTTATTGTATTC-3′). An *Eco*RI site was incorporated at the 5′ end and a *Kpn*I site at the 3′ end, and whole NOX4 PCR fragment was cloned into a pCMV-HA vector (Clontech, Palo Alto, CA; cat. no. 631604). The constructs were transformed into DH10α-competent cells and subsequently isolated using E.Z.N.A Fastfilter Midi kit. The constructs were sequenced by MWG-Biotech (Milton-Keynes, UK). NIH3T3 fibroblasts were mock-transfected (transfection medium only) or transfected with the NOX4 construct using Lipofectamine 2000 (see above) or L-PEI. In the latter case, 3 μg of plasmid was mixed with 150 mM NaCl to a final volume of 100 μl and 9.6 μl L-PEI was mixed with 150 mM NaCl to a final volume of 100 μl. The L-PEI solution was added to the plasmid solution during vortexing. The final solution was mixed for 15 s and left at room temperature for 20 min Growth medium was changed to 1,300 μl DMEM without serum or antibiotics. Plasmid solution (200 μl) was added per well dropwise. Cells were left with the plasmid solution for 3–4 h before changing medium back to DMEM with serum. Cells were left for transfection 48 h before experimental use.

#### Estimation of ROS Production

ROS production was estimated as previously described (Hansen et al. 2011). Cells grown on pretreated coverslips were preincubated in serum-free growth medium containing the ROS-sensitive fluorescent probe carboxy-H2DCFDA (25 lM, 2 h). Coverslips were washed with iso-osmotic NaCl medium and placed in iso-osmotic, hyperosmotic or hypo-osmotic NaCl medium. ROS was estimated at 37°C on a thermostatic PTI (Princeton, NJ) Ratio Master spectrophotometer using excitation and emission wavelengths of 490 and 515 nm, respectively. ROS production was estimated from the initial increase in fluorescence from 0 to 20 s.

#### Western Blotting

Cell lysates were prepared in lysis buffer (1% SDS, 150 mM NaCl, 20 mM HEPES, 1 mM EDTA, 0.5% Triton X-100, 1 mM NaVO_3_, and 1% protease inhibitor mix). SDS-PAGE gel electrophoresis was carried out in 10% Bis–Tris gels using NuPAGE MOPS SDS running buffer (Invitrogen). Proteins were transferred to nitrocellulose membranes using NuPAGE transfer buffer (Invitrogen) and the membranes blocked in TBST (0.01 M Tris–HCl, 0.15 M NaCl, 0.1% Tween 20, pH 7.4) containing 5% nonfat dry milk. Proteins were probed with antibodies against NOX4 (Novus Biologicals, Littleton, CO) or histone H3 (Santa Cruz Biotechnology, Santa Cruz, CA). Protein–antibody complexes were visualized using BCIP/NBT (KPL, Gaithersburg, MD).

#### MTT Assay—Cell Viability

The MTT calorimetric assay was used to estimate the ability of cells to convert the yellow soluble tetrazolium salt 3-(4,5-dimethylthiazol-2-yl)-2,5-diphenyltetrazolium bromide (MTT) into a blue insoluble formazan precipitate. Cells were seeded in 96-well microplates (16 × 10^3^ in 200 μl medium) and incubated overnight (37°C, 5% CO_2_). H_2_0_2_ was added, and cells were incubated for 4 h. The MTT solution (5 mg/ml sterilized PBS) was added (25 μl/well) and the plate incubated (37°C, 5% CO_2_) for 3 h. One hundred microliters of SDS–HCl solution (5 ml 0.01 M HCl, 0.5 g SDS) was added to each well and mixed to lyse the cells and solubilize the colored formazan crystals. Samples were measured at 570 nm using a FLUOstar OPTIMA 96-well microplate plate reader (BMG Lab Technologies, Offenburg, Germany). Data are reported in terms of relative cell viability compared to control cells with no H_2_O_2_. Absorbance values were assumed to be directly proportional to the number of viable cells. Each experiment was performed in triplicate.

#### Statistics

All data are presented either as individual experiments or as mean values ± standard error of the mean (SEM). Statistical evaluation is based on two-way ANOVA or a Student’s *t*-test (specified in legends).

## Results

### Taurine Uptake is Reduced by Osmotic Cell Swelling *per se*

**Fig. 1:**
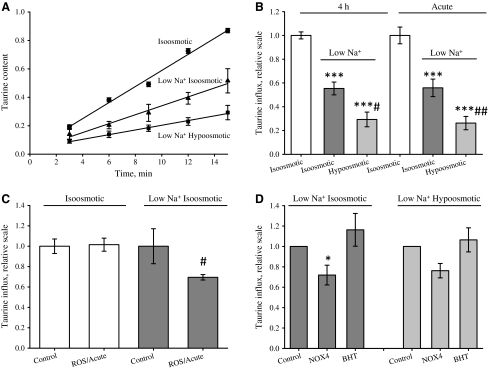
The reduction in taurine uptake following hypo-osmotic exposure is independent of NOX4 activity. Taurine uptake (nmol g protein^−1^) was estimated by the tracer technique in NIH3T3 cells exposed to either 4 h iso-osmotic DMEM, low Na^+^ hypo-osmotic DMEM (200 mOsm) and low Na^+^ iso-osmotic DMEM (335 mOsm, adjusted to osmolarity by addition of sucrose) or acutely (*Acute*) to iso-osmotic NaCl, low Na^+^ hypo-osmotic NaCl and low Na^+^ iso-osmotic NaCl (335 mOsm, adjusted to osmolarity by addition of sucrose), as described in Materials and Methods. Taurine influx (nmol g protein^−1^ min^−1^) was estimated by linear regression of taurine uptake within 15 min. **a** Taurine uptake following 4 h incubation in the respective DMEM solutions. Data represent three sets of paired experiments. **b** Taurine influx (4 h and acute) in cells exposed for 4 h to the DMEM solutions (4 h) or acutely to the NaCl medium. Absolute values for controls are 0.056 ± 0.002 nmol g protein^−1^ min^−1^ (4 h, *n* *=* 3) and 0.222 ± 0.019 nmol g protein^−1^ min^−1^ (acute, *n* = 4). Significance was determined using two-way ANOVA with Bonferroni post test, comparing treatments within the acute and 4 h groups, respectively. **c** Taurine influx in cells exposed acutely to iso-osmotic and low Na^+^ iso-osmotic NaCl with or without acute exposure to 0.5 mM H_2_O_2_. Absolute values for controls are 0.230 ± 0.016 nmol g protein^−1^ min^−1^ (iso-osmotic, *n* = 3) and 0.089 ± 0.015 nmol g protein^−1^ min^−1^ (low Na^+^ iso-osmotic, *n* = 3). Significance was determined with Student’s *t*-test (paired, one-sided) comparing influx with and without ROS/acute with the respective control. **d** Taurine influx estimated in cells acutely exposed to low Na^+^ iso-osmotic or low Na^+^ hypo-osmotic NaCl. NOX4 overexpression was carried out as described in Materials and Methods. BHT (0.5 mM) was present during the influx experiments. Statistical evaluation by two-way ANOVA with Bonferroni post test comparing influx from each treatment with the respective control; e.g., BHT-treated cells were compared with untreated, whereas NOX4-overexpressing cells were compared with mock-treated cells under iso-osmotic and hypo-osmotic conditions. All values are given relative to the respective control ± SEM. *P < 0.05, **P < 0.01, ***P < 0.001 compared to the respective control; ^#^ *P* < 0.05, ^##^ *P* < 0.01 compared to low Na^+^ iso-osmotic

Taurine uptake in NIH3T3 mouse fibroblasts was previously shown to be totally Na^+^-dependent and eliminated in the presence of the taurine analog β-alanine (Voss et al. 2004), indicating that taurine uptake in the fibroblasts is mediated by TauT. From Fig. 1a, b it is seen that reduction in the total extracellular Na^+^ concentration from 150 to 80 mM for 4 h, keeping osmolarity constant with sucrose, results in a significant reduction in the taurine influx in NIH3T3 cells to 56% of the iso-osmotic value (compare “iso-osmotic” to “low Na^+^ iso-osmotic”). From Fig. 1a, b it is also seen that reduction in the extracellular osmolarity from 335 to 200 mOsm, keeping the extracellular Na^+^ concentration constant at 80 mM, leads to an additional 50% reduction in taurine uptake (compare “low Na^+^ iso-osmotic” to “low Na^+^ hypo-osmotic”). Hence, TauT activity is reduced by 4 h exposure to hypo-osmotic conditions due to reduction in the extracellular Na^+^ concentration as well as reduction in the extracellular osmolarity, i.e., osmotic cell swelling. This is similar to observations in Ehrlich ascites tumor cells (Hoffmann and Lambert 1983). To test whether reduced expression or membrane localization of TauT is responsible for the decreased taurine uptake under long-term hypo-osmotic conditions (4 h), we compared taurine uptake in cells exposed to 4 h reduction in the extracellular osmolarity with cells exposed acutely to hypo-osmotic conditions. From Fig. 1b it is seen that taurine uptake is reduced to the same extent following 4 h and acute reduction in the extracellular Na^+^ concentration (compare dark gray bars at 4 h and acute) and in extracellular osmolarity (compare light gray bars at 4 h and acute). As acute and 4 h reduction in Na^+^ and osmolarity give the same reduction in influx, it is suggested that the reduction in taurine uptake is most likely caused by direct inhibition of TauT. Similarly, it was previously shown in Ehrlich ascites cells that the regulation of the activity of another osmoregulatory transporter, NKCC1, by changes in osmolarity is not related to the number of transport molecules present in the membrane (Hoffmann et al. 1986).

### ROS Reduce Taurine Uptake under Conditions with Low Extracellular Na^+^

Protein phosphorylation is modulated by ROS as protein phosphatases contain a redox-sensitive cysteine group in the catalytic site, rendering them inactive when oxidized by otherwise weak oxidants, such as H_2_O_2_ (Sommer et al. 2002; Meng et al. 2002; Wright et al. 2009; Barchowsky et al. 1995; Lee et al. 1998). ROS production in NIH3T3 cells is increased under hyperosmotic (Supplementary Fig. 1) as well as hypo-osmotic (Supplementary Fig. 2) conditions, and we speculated whether the reduced taurine uptake following hypo-osmotic cell swelling (Fig. 1b) reflects a ROS-induced shift in TauT’s Na^+^ sensitivity. From Supplementary Fig. 3a and Fig. 1c it is seen that acute exposure to 0.5 mM H_2_O_2_ has no detectable effect on taurine uptake under either hyperosmotic or iso-osmotic conditions, respectively, whereas 0.5 mM H_2_O_2_ results in a significant reduction in taurine influx under iso-osmotic conditions with low extracellular Na^+^ concentration. It is emphasized that the effect of ROS in the latter case was tested in a medium with low Na^+^ concentration, which was supplemented to isotonicity with sucrose in order to avoid taurine influx via the swelling-induced and ROS-sensitive taurine release pathway (Hansen et al. 2011; Lambert 2007). Exposure to 2 mM H_2_O_2_ was previously reported to reduce taurine uptake in NIH3T3 cells under iso-osmotic conditions (Voss et al. 2004). However, long-term exposure (4 h) to 0.5 mM H_2_O_2_ is accompanied by a reduction in taurine uptake as well as cell survival; i.e., acute exposure to a high dose or long-term exposure to a low dose of H_2_O_2_ is likely to kill NIH3T3 cells (Supplementary Fig. 3b).

Kinetic analysis of taurine uptake versus the extracellular Na^+^ concentration (medium supplemented to isotonicity with NMDG) revealed that acute exposure to 0.5 mM H_2_O_2_ increased the Na^+^:taurine stoichiometry significantly by 17 ± 7% (control 1.93 ± 0.14, H_2_O_2_-treated 2.24 ± 0.06, *n* = 4), whereas it had no significant effect on the TauT affinity for Na^+^ (control 81 ± 13 mM, H_2_O_2_-treated 72 ± 2 mM, *n* = 4) or the maximal transport rate (H_2_O_2_ relative to control 0.94 ± 0.11, *n* = 4). ROS is generated by NOX4 under hypo-osmotic conditions in NIH3T3 cells (Supplementary Fig. 2), and in order to determine whether ROS produced by NOX4 could mimic the effect of acute exposure to H_2_O_2_ and cause a reduction in TauT taurine transport under conditions with low extracellular Na^+^ concentration, we used ROS scavenging by butylated hydroxytoluene (BHT) (Lambert 2003) and overexpression of NOX4. There is a roughly similar reduction in taurine uptake with ROS acute and NOX4 (compare Fig. 1c, d). ROS scavenging with BHT has no significant effect on taurine uptake at low extracellular Na^+^ concentration under iso-osmotic and hypo-osmotic conditions (Fig. 1d), indicating that ROS scavenging has no effect on taurine uptake at low extracellular Na^+^ concentrations.

### TauT mRNA is Reduced under Hypo-Osmotic Conditions

**Fig. 2:**
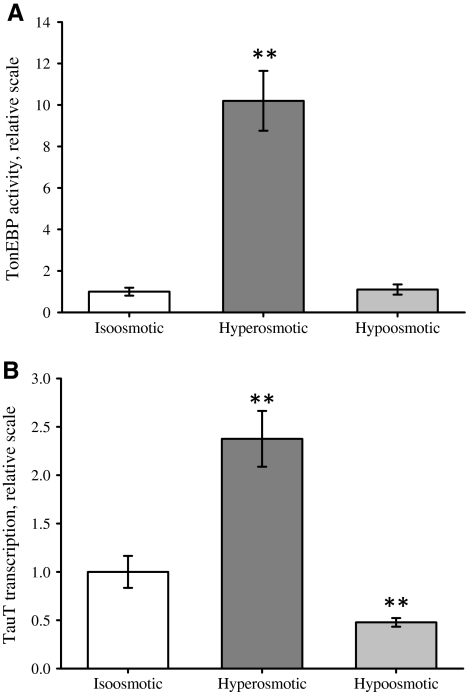
Effect of long-term hyper- and hypo-osmotic conditions on TonEBP activity and TauT transcription. TonEBP activity and TauT transcription were estimated in NIH3T3 cells exposed to iso-osmotic, hyperosmotic or hypo-osmotic medium (DMEM) for 16 and 4 h, respectively. **a** For estimation of TonEBP activity, cells were transfected with luciferase-plasmid and incubated with the indicated medium and luciferase activity was estimated as indicated in Materials and Methods. **b** TauT mRNA transcription was estimated by qPCR. cDNA was generated from mock-transfected NIH3T3 cells, and qPCR was performed using primers specific for TauT mRNA as indicated in Materials and Methods. Values are given relative to their respective iso-osmotic control ± SEM. Data in (**a**) represent seven sets of paired experiments. Data in (**b**) represent four and five sets of paired experiments for hyperosmotic and hypo-osmotic, respectively. Statistical evaluation for (**a**) and (**b**) by Student’s *t*-test (paired, one-sided) comparing hyperosmotic or hypo-osmotic to iso-osmotic control, respectively. *P < 0.05, **P < 0.01 compared to iso-osmotic control

TauT transcription in mammalian cells is upregulated by TonEBP under hyperosmotic conditions (Miyakawa et al. 1998, 1999b), and we have previously shown that 4 h hyperosmotic exposure increases TauT activity in, e.g., NIH3T3 cells (Voss et al. 2004). As the effects of hypo-osmotic exposure on taurine uptake appeared to be acute and independent of TauT transcription, we tested whether TonEBP activity and TauT transcription were actually unaffected by prolonged exposure to hypo-osmotic conditions. From Fig. 2 it is seen, in accordance with previous data, that exposure of NIH3T3 cells to hyperosmotic conditions results in a significant 16-fold increase in TonEBP activity within 16 h (Fig. 2a) and an almost twofold increase in TauT mRNA within 4 h (Fig. 2b). However, TonEBP activity is unaffected by 16 h hypo-osmotic exposure (Fig. 2a), which is in agreement with previously demonstration of a reduction in TonEBP mRNA and retention of TonEBP in the cytoplasm under hypo-osmotic conditions (Woo et al. 2000). However, despite the unaffected TonEBP activity, TauT mRNA is reduced after 4 h hypo-osmotic exposure (Fig. 2b). A selection of cells expressing low TauT as the cause of the observed data is most unlikely as kinetic analysis (Voss et al. 2004) revealed that there is only one population of NIH3T3 cells (one *K*_m_ value for taurine) and that the time frame of our experiments is very short. Hence, TauT mRNA levels correlate with TonEBP activity under long-term hyperosmotic conditions but not under long-term hypo-osmotic conditions.

**Fig. 3:**
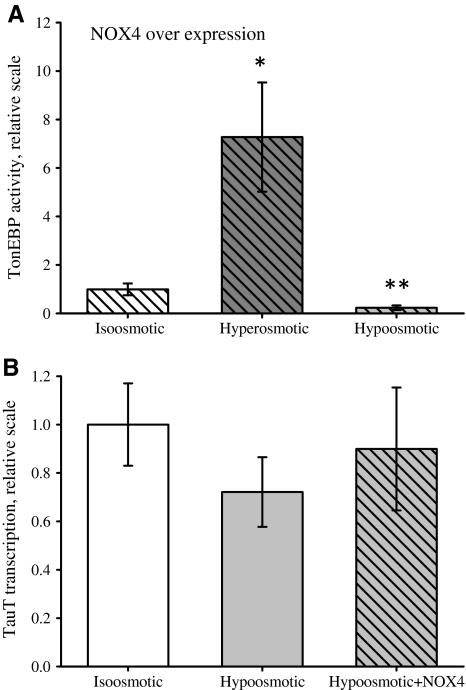
NOX4 regulates TonEBP activity, but not TauT transcription, under hypo-osmotic conditions. TonEBP activity and TauT transcription were estimated in NIH3T3 cells exposed for 4 h to iso-osmotic or hypo-osmotic medium (DMEM). Cells were transfected with NOX4 (*hatched bars*) or mock-transfected (*open bars*) as described in Materials and Methods. TonEBP activity and TauT mRNA were estimated as described in the legend to Fig. 2 and Materials and Methods. **a** TonEBP activity in NOX4-transfected cells relative to non-transfected iso-osmotic control. **b** TauT mRNA transcription in mock- and NOX4-transfected cells. Values are given relative to their respective iso-osmotic control ± SEM. Data in (**a**) represent seven sets of experiments. Data in (**b**) represent four (hypo-osmotic) and three (NOX4) sets of experiments. Statistical evaluation for (**a**) and (**b**) by Student’s *t*-test (paired, one-sided) comparing hyperosmotic, hypo-osmotic or hypo-osmotic+NOX4 to the respective iso-osmotic control. Values are given relative to iso-osmotic control ± SEM. *P < 0.05, **P < 0.01 compared to iso-osmotic control

Using NOX4-overexpressing cells, we tested whether ROS generated by NOX4 could affect TonEBP activity and TauT transcription. It is seen that the increase in TonEBP activity following hypertonic exposure in NOX4-overexpressing cells is comparable to that in native cells, whereas there is a significant decrease in TonEBP activity under hypo-osmotic conditions (4 h) (Fig. 3a). The significant reduction in TonEBP activity under hypo-osmotic conditions compared to iso-osmotic conditions is taken to indicate that TonEBP is negatively regulated by excess ROS produced by NOX4 under hypo-osmotic conditions. From Fig. 3b it is seen that TauT mRNA transcription is unaltered under hypo-osmotic conditions when overexpressing NOX4. Thus, the increased ROS availability generated from NOX4 under hypo-osmotic conditions does not seem to suppress *TauT* transcription via the altered TonEBP activity.

To test these results, we applied ROS (H_2_O_2_) and the phosphatase inhibitor vanadate and estimated TonEBP activity. Unexpectedly, we found that both ROS and vanadate reduced TonEBP activity significantly when added acutely to NIH3T3 cells preincubated under hyperosmotic conditions (16 h, 500 mOsm), i.e., immediately before lysis and estimation of luciferase activity (Supplementary Fig. 4a). Mg^2+^-dependent ATPases are inhibited by vanadate (Bond and Hudgins 1980; Hanocq-Quertier et al. 1988), and as the luciferase used in this assay requires Mg^2+^ as cosubstrate (Promega technical bulletin 281), the observed acute effect of vanadate on the TonEBP assay is most likely a result of luciferase inhibition. Thus, H_2_O_2_ and vanadate most likely interfere directly with luciferase activity. Furthermore, prolonged exposure to H_2_O_2_ (0.5 mM) under hyperosmotic conditions results in significantly reduced TauT transcription (Supplementary Fig. 4b); this is, however, most likely an effect of the significant cell death following prolonged exposure to H_2_O_2_ (Supplementary Fig. 3b). The effect of H_2_O_2_ and vanadate on TonEBP activity and TauT transcription will therefore not be discussed further.

**Fig. 4:**
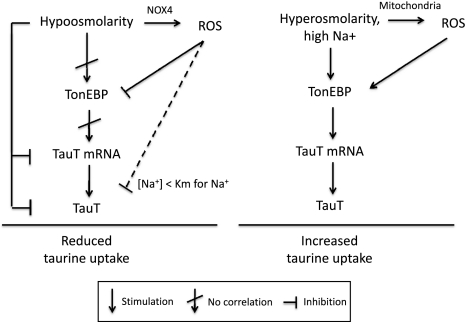
Modulation of taurine uptake by TauT following osmotic stress. The model is described in the text

## Discussion

Active taurine uptake in mammalian cells is fueled by the prevailing Na^+^ gradient, and TauT activity is regulated by various protein kinases, e.g., protein kinase A (PKA) and PKC plus casein kinase 2 (CK2) (Hansen et al. 2011; Jacobsen et al. 2008; Voss et al. 2004). We find here, in agreement with previous results (Hoffmann and Lambert 1983), that osmotic cell swelling per se results in an inhibition of taurine uptake. Acute regulation of TauT often involves a shift in the maximal transport capacity (*V*_max_), the substrate concentration required for half-maximal transport activity (*K*_m_ values for taurine [*K*_m taurine_] and Na^+^ [*K*_m Na_]) and/or the Na^+^:taurine transport stoichiometry (Lambert 2004). In the case of NIH3T3 fibroblasts, we recently demonstrated that phosphorylation, mediated by the constitutively active serin/threonine kinase CK2, reduces the affinity of TauT toward Na^+^ plus the maximal transport activity and increases the Na^+^:taurine stoichiometry and that the effect of CK2 inhibition on TauT activity was more pronounced at an extracellular Na^+^ concentration close to *K*_m Na_ (Hansen et al. 2011). Osmotic cell swelling is accompanied by an increase in the production of ROS in NIH3T3 mouse fibroblasts, porcine myotubes, HTC cells (liver-derived cell line), HEK293 cells, collecting duct cells and neonatal rat cardiomyocytes (Zhou et al. 2006; Diaz-Elizondo et al. 2006; Friis et al. 2008; Lambert 2003; Varela et al. 2004; Ørtenblad et al. 2003; Hansen et al. 2011); and we have recently shown that a NOX4/p22phox complex constitutes the catalytic core of the volume-sensitive NADPH oxidase in NIH3T3 fibroblasts (Friis et al. 2008). Furthermore, NADPH-oxidase activity has been assigned a role in the increased ROS production during hyponatremia (Haussinger and Schliess 2008). It thus seemed likely that taurine uptake could be modulated by ROS following hypo-osmotic/hyponatremic conditions.

The present data indicate that acute exposure to 0.5 mM H_2_O_2_ under iso-osmotic conditions has no detectable effect on taurine uptake at standard extracellular Na^+^ concentration, whereas H_2_O_2_ reduces uptake under conditions with low extracellular Na^+^ (Fig. 1c). NOX4 overexpression increases the swelling-induced ROS production in NIH3T3 cells and, similar to H_2_O_2_ exposure, appears to reduce taurine uptake under conditions with low extracellular Na^+^ concentration (Fig. 1c, d). We also find that the Na^+^:taurine transport stoichiometry is increased by addition of 0.5 mM H_2_O_2_; i.e., lower concentrations of Na^+^ are required for equivalent taurine uptake following addition of H_2_O_2_ as long as the concentration of sodium is above *K*_m Na_. Increased availability of ROS under hypo-osmotic conditions can partly explain the observed reduction in taurine uptake when the extracellular Na^+^ concentration is reduced. However, the increased Na^+^:taurine stoichiometry by ROS will only reduce the uptake of taurine at Na^+^ concentrations below *K*_m Na_. As the Na^+^ concentration is not reduced below *K*_m Na_ even under severe hyponatremia, this effect is probably not pathophysiologically relevant.

### ROS—TonEBP—Tonicity Sensitivity

Long-term regulation in NIH3T3 cells, following continuous exposure to hyperosmotic stress or substrate (taurine), relies on the transcriptional modulation of the gene encoding TauT (Voss et al. 2004). The transcriptional increase in the expression of the Na^+^-dependent co-transporters, sodium/*myo*-inositol co-transporter (SMIT), sodium/chloride/betaine co-transporter (BGT1), and TauT is facilitated by the *cis*-element tonicity-response enhancer (TonE) (Miyakawa et al. 1998; Rim et al. 1998), which is regulated by TonEBP (Han et al. 2006; Ito et al. 2004; Jeon et al. 2006; Miyakawa et al. 1999b). TonEBP plays a key role in the protection of cells from prolonged increase in the extracellular osmolarity by increasing the cellular content of osmolytes through transcription of co-transporters for organic and inorganic osmolytes (Han et al. 2006; Ito et al. 2004; Jeon et al. 2006; Miyakawa et al. 1999a, 1999b). Mice lacking functional TonEBP (TonEBP^−/−^) have a severe renal medulla degeneration caused by low levels of SMIT, aldose reductase and TauT expression (Lopez-Rodriguez et al. 2004). TonEBP is evenly distributed between the cytosol and nucleus under iso-osmotic conditions, whereas hyperosmolarity increases and hypo-osmolarity decreases the nuclear fraction (Woo et al. 2000; Miyakawa et al. 1999b; Cha et al. 2001; Tong et al. 2006). In accordance, hyperosmotic stress results in increased expression and activation of TonEBP (Lopez-Rodriguez et al. 2004; Woo et al. 2002; Zhou et al. 2006; Cai et al. 2005), whereas TonEBP transcription is reduced and nuclear export accelerated during hypo-osmotic stress (Woo et al. 2000; Tong et al. 2006). TonEBP activity and nuclear translocation are regulated by serine and tyrosine phosphorylation (reviewed in Burg et al. 2007; Aramburu et al. 2006). The increased TonEBP activity following hyperosmotic exposure involves mitochondrial release of ROS (Zhou et al. 2005, 2006; Ferraris et al. 2002). In the present study we demonstrate that TonEBP activity is similarly stimulated under hyperosmotic conditions but unaffected under hypo-osmotic conditions (Fig. 2). In NOX4-overexpressing NIH3T3 cells TonEBP activity is still increased under hyperosmotic conditions, whereas TonEBP activity is significantly reduced under hypo-osmotic conditions (Fig. 3).

Figure 4 summarizes data and illustrates modulation of taurine uptake by TauT in NIH3T3 cells following osmotic stress. TauT transcription is generally assumed to follow TonEBP activity. This is also the case for TauT transcription under hyperosmotic conditions (Fig. 2). However, we find that under long-term hypo-osmotic exposure the downregulation of TauT transcription is not secondary to reduced TonEBP activity as (1) TonEBP activity is unaltered whereas TauT mRNA is reduced (Fig. 2) and (2) stimulation and hindrance of TonEBP activity by increased NOX4 expression do not correlate with TauT transcription level (Fig. 3). The lack of correlation between TonEBP activity and TauT transcription under hypo-osmotic conditions could indicate that TauT transcription is dependent on other transcription factors inactivated by hypo-osmolarity. Under hyperosmotic conditions, ROS generated from the mitochondria are reported to stimulate TonEBP transactivation via a hyper-osmotically induced transactivation domain (TAD) (Zhou et al. 2006), and we hypothesize that the primary effect of ROS on TonEBP-TAD is an increased sensitivity toward tonicity. In this scenario ROS stimulate TonEBP transactivation under conditions with high extracellular ion concentrations, whereas TonEBP transactivation is further suppressed by NOX4-generated ROS under conditions with low extracellular ion concentrations. Our current hypothesis is that ROS-mediated interference with TauT kinetics is only visible under conditions with very low Na^+^ concentrations, i.e., concentrations significantly lower than those observed under hypo-osmotic hyponatremia, but will have no effect on taurine transport under conditions with high or normal extracellular Na^+^ concentrations. The hypo-osmotically induced reduction in TauT mRNA will reduce TauT activity following prolonged osmotic stress. The reduction in cellular taurine content following hyponatremia is probably dominated by increased taurine leak, whereas more chronic conditions can involve the reduction in TauT mRNA.

## Electronic supplementary material

Below is the link to the electronic supplementary material.

Figure S1 Generation of ROS under hyperosmotic conditions. Time-trace of ROS-production in NIH3T3 cells exposed acutely to isoosmotic or hyperosmotic conditions. ROS production was estimated in NIH3T3 cells under acute hypoosmotic conditions using the ROS-sensitive probe carboxy-H_2_DCFDA as described in materials and methods (PDF 64 kb)

Figure S2 Modulation of hypoosmotic ROS-production following NOX4-overexpression. NIH3T3 cells were Transfected for 48 h with NOX4-plasmid and ROS-production estimated as described in Materials and Methods.** A**: Time-trace of ROS-production in mock-transfected cells under isoosmotic/hypoosmotic conditions, and NOX4 transfected cells under hypoosmotic conditions.** A**,** Inset**: Westernblot of control and NOX4 overexpressing cells as described in materials and methods.** B**: Quantification of ROS-production under isoosmotic (open bars) and hypoosmotic (grey bars) exposure in NOX4-transfected cells compared to mock control. The ROS-production was estimated as the initial slope (0-20 sec) of time traces illustrated in A and data represent 4 sets of paired experiments. Values are given relative to the respective control ± SEM. Level of significance: # P < 0.05 compared to Hypoosmotic control (PDF 92 kb)

Figure S3 The effect of H_2_O_2_ on taurine uptake under hyperosmotic conditions and cell viability. Taurine uptake and MTT-assay as indicated in materials and methods in NIH3T3 cells.**A**: Taurine uptake was estimated in NIH3T3 cells following 4 hours preincubation in hypertonic (500 mOsm) solutions. H_2_O_2_ (0.5 mM) was present during estimation of the taurine influx only (acute) or during the preincubation plus the subsequent influx estimation (4 h). Data represent 3 sets of paired experiments.** B**: Cell survival was estimated by The MTT calorimetric assay on cells exposed to no (Control) or 0.2 mM / 0.5 mM H_2_O_2_ for 4 hours. Values are given relative to the respective control ± SEM. Level of significance: * P < 0.05, ** P < 0.01 compared to Control(PDF 24 kb)

Figure S4 Effect of acute ROS and vanadate on TonEBP activity and long term exposure to ROS on* TauT* transcription under hyperosmotic conditions. TonEBP activity and TauT transcription was estimated in cells exposed to isoosmotic or hyperosmotic media (DMEM) for 16 and 4 hours, respectively. Estimation as indicated in materials and methods and Figure 2. Data for TonEBP represent 7, 4 and 4 sets of experiments for Hyperosmotic, ROS/Acute and Vanadate/Acute, respectively. Data for TauT transcription represent 4 sets of experiments. Values are given relative to Isoosmotic control ± SEM. Level of significance: * P < 0.05 compared to Isoosmotic control, # P < 0.05 compared to Hyperosmotic control (PDF 21 kb)
