# Supplementary figures and images for: Downregulation of the Taurine Transporter TauT During Hypo-Osmotic Stress in NIH3T3 Mouse Fibroblasts
Source: J Membr Biol. 2012 Mar 2;245(2):77–87. doi: 10.1007/s00232-012-9416-8 (PMC3298736; doi:10.1007/s00232-012-9416-8)

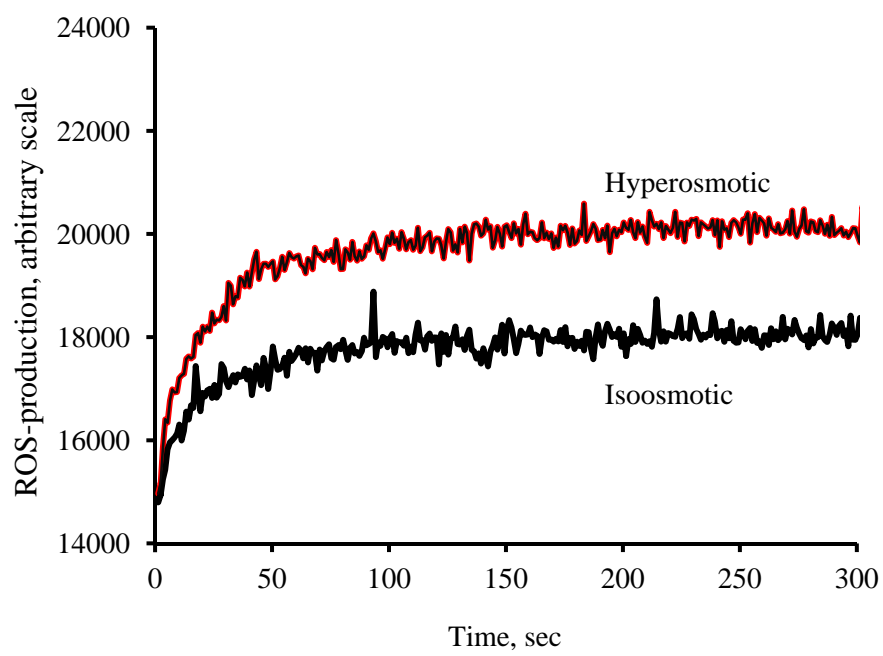

Fig S1

Supplement: Supplementary file 1 — Generation of ROS under hyperosmotic conditions. Time-trace of ROS-production in NIH3T3 cells exposed acutely to isoosmotic or hyperosmotic conditions. ROS production was estimated in NIH3T3 cells under acute hypoosmotic conditions using the ROS-sensitive probe carboxy-H2DCFDA as described in materials and methods (PDF 64 kb) [file 232_2012_9416_MOESM1_ESM.pdf]

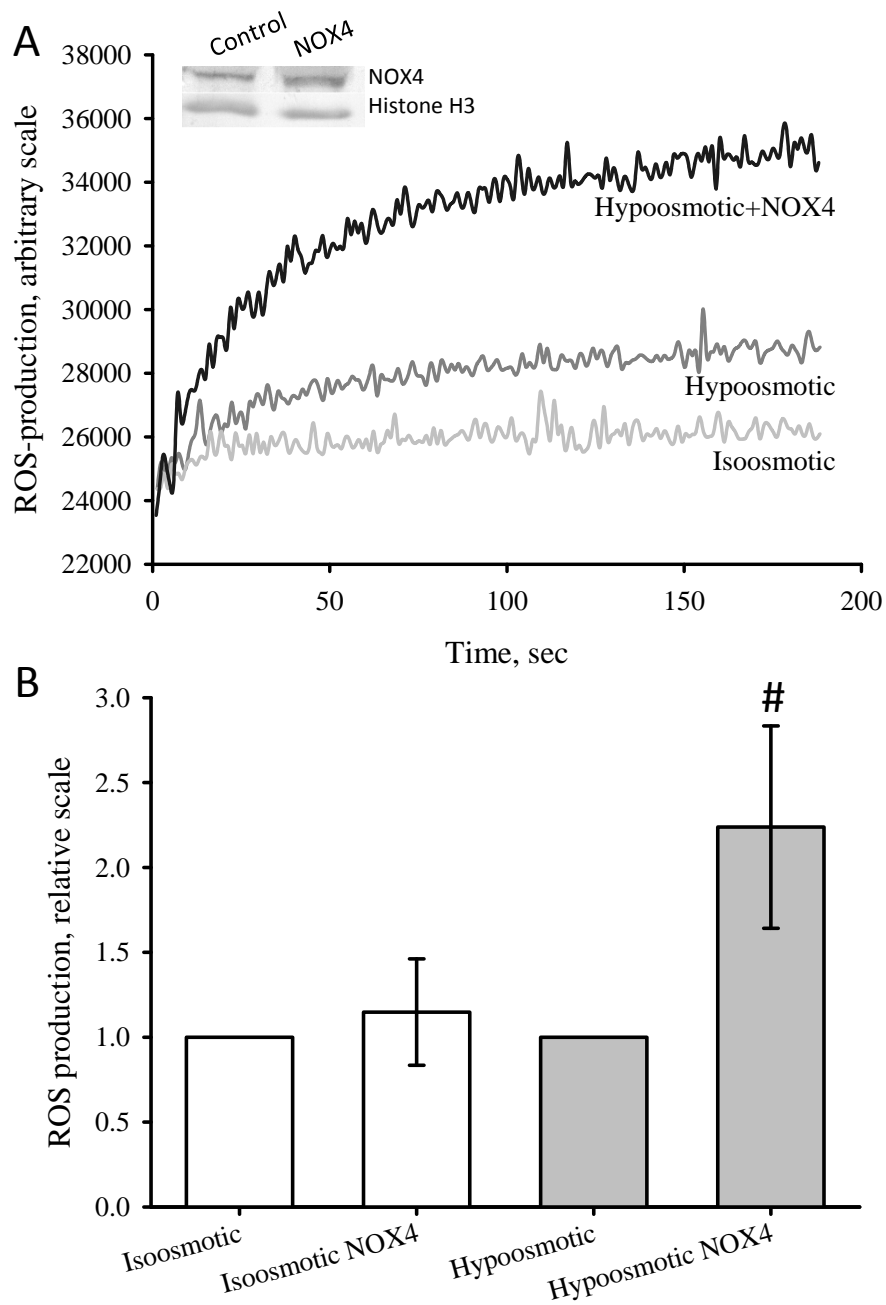

Fig S2

Supplement: Supplementary file 2 — Modulation of hypoosmotic ROS-production following NOX4-overexpression. NIH3T3 cells were Transfected for 48 h with NOX4-plasmid and ROS-production estimated as described in Materials and Methods. A: Time-trace of ROS-production in mock-transfected cells under isoosmotic/hypoosmotic conditions, and NOX4 transfected cells under hypoosmotic conditions. A, Inset: Westernblot of control and NOX4 overexpressing cells as described in materials and methods. B: Quantification of ROS-production under isoosmotic (open bars) and hypoosmotic (grey bars) exposure in NOX4-transfected cells compared to mock control. The ROS-production was estimated as the initial slope (0-20 sec) of time traces illustrated in A and data represent 4 sets of paired experiments. Values are given relative to the respective control ± SEM. Level of significance: # P < 0.05 compared to Hypoosmotic control (PDF 92 kb) [file 232_2012_9416_MOESM2_ESM.pdf]

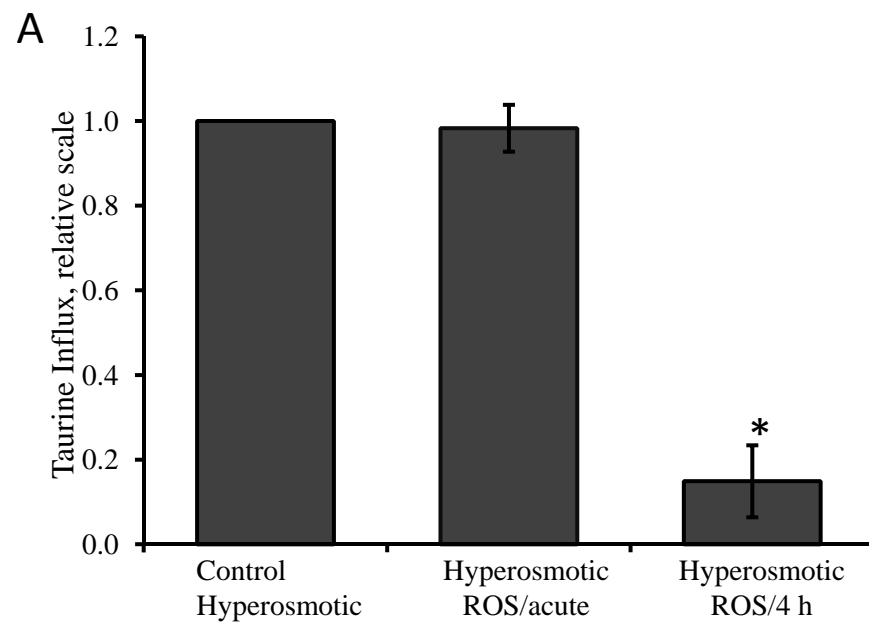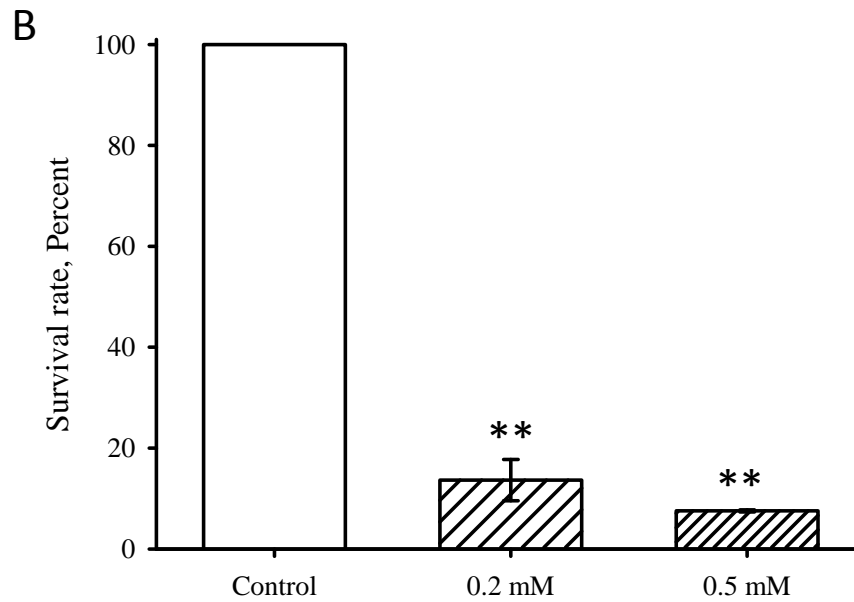

Fig S3

Supplement: Supplementary file 3 — The effect of H2O2 on taurine uptake under hyperosmotic conditions and cell viability. Taurine uptake and MTT-assay as indicated in materials and methods in NIH3T3 cells.A: Taurine uptake was estimated in NIH3T3 cells following 4 hours preincubation in hypertonic (500 mOsm) solutions. H2O2 (0.5 mM) was present during estimation of the taurine influx only (acute) or during the preincubation plus the subsequent influx estimation (4 h). Data represent 3 sets of paired experiments. B: Cell survival was estimated by The MTT calorimetric assay on cells exposed to no (Control) or 0.2 mM / 0.5 mM H2O2 for 4 hours. Values are given relative to the respective control ± SEM. Level of significance: * P < 0.05, ** P < 0.01 compared to Control(PDF 24 kb) [file 232_2012_9416_MOESM3_ESM.pdf]

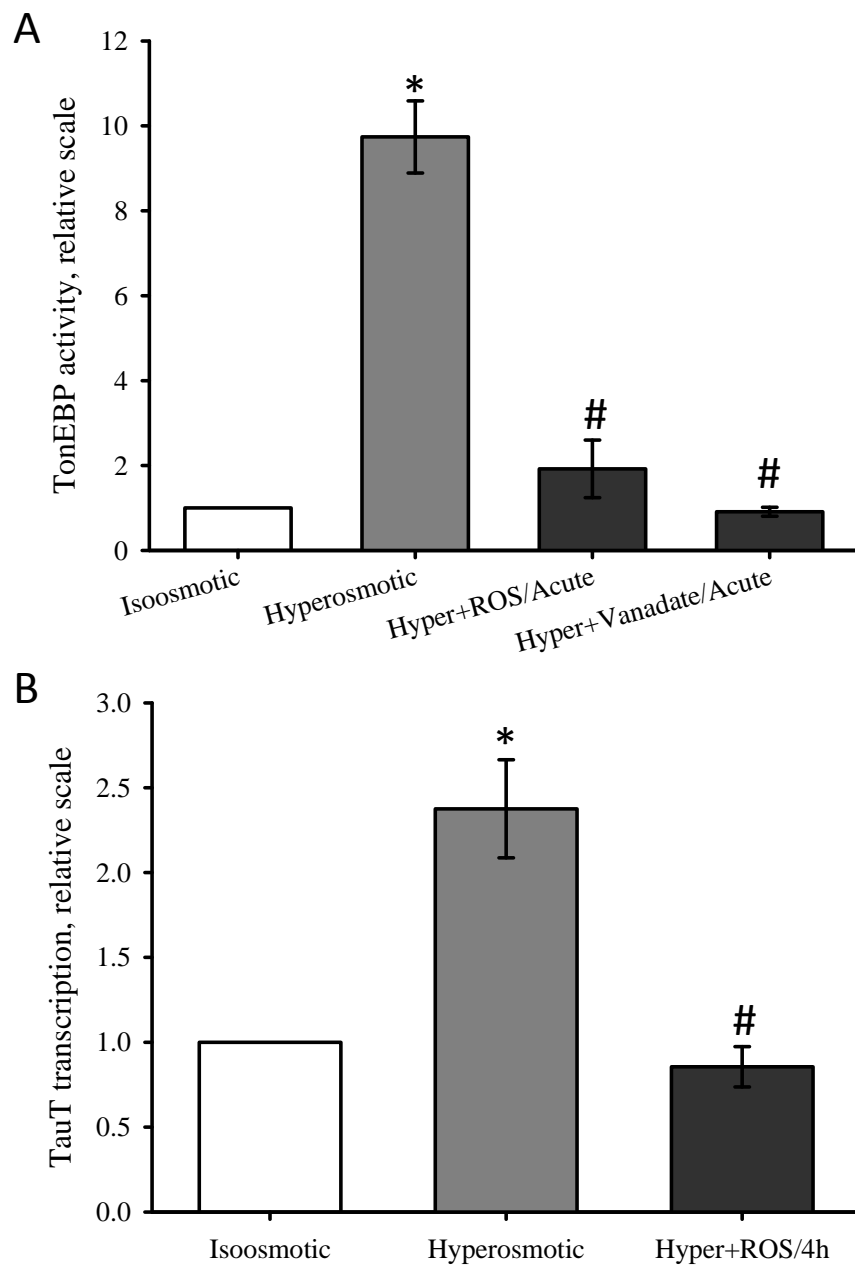

Fig S4

Supplement: Supplementary file 4 — Effect of acute ROS and vanadate on TonEBP activity and long term exposure to ROS on TauT transcription under hyperosmotic conditions. TonEBP activity and TauT transcription was estimated in cells exposed to isoosmotic or hyperosmotic media (DMEM) for 16 and 4 hours, respectively. Estimation as indicated in materials and methods and Figure 2. Data for TonEBP represent 7, 4 and 4 sets of experiments for Hyperosmotic, ROS/Acute and Vanadate/Acute, respectively. Data for TauT transcription represent 4 sets of experiments. Values are given relative to Isoosmotic control ± SEM. Level of significance: * P < 0.05 compared to Isoosmotic control, # P < 0.05 compared to Hyperosmotic control (PDF 21 kb) [file 232_2012_9416_MOESM4_ESM.pdf]
